# Lifting the veil on amyloid drug design

**DOI:** 10.7554/eLife.01089

**Published:** 2013-07-16

**Authors:** Kathryn E Tiller, Peter M Tessier

**Affiliations:** 1 **Kathryn E Tiller** is in the Center for Biotechnology & Interdisciplinary Studies, Department of Chemical and Biological Engineering, Rensselaer Polytechnic Institute, New York, United Statestillek@rpi.edu; 2 **Peter M Tessier** is in the Center for Biotechnology & Interdisciplinary Studies, Department of Chemical and Biological Engineering, Rensselaer Polytechnic Institute, New York, United Statestessier@rpi.edu

**Keywords:** amyloid fiber, computational biology, drug discovery, Alzheimer's disease, ligand docking, Other

## Abstract

High resolution structures and computational methods have been used to identify compounds that prevent amyloid fibrils associated with Alzheimer’s disease from dissociating into toxic species.

**Related research article** Jiang L, Liu C, Leibly D, Landau M, Zhao M, Hughes MP, Eisenberg DS. 2013. Structure-based discovery of fiber-binding compounds that reduce the cytotoxicity of amyloid beta. *eLife* **2**:e00857. doi: 10.7554/eLife.00857

**Image** The structure of amyloid has been used to guide the design of compounds (blue) that reduce amyloid toxicity

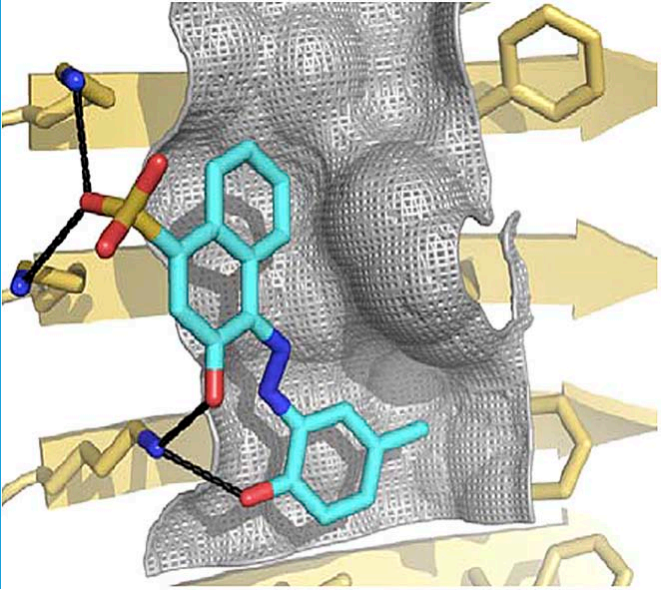

Some of the most debilitating human disorders—including Alzheimer’s, Parkinson’s and Huntington’s diseases—are marked by the inappropriate assembly of peptides and proteins into particles of various sizes, structures and toxicities. These range from highly toxic small oligomers, each consisting of a few monomers, to large insoluble aggregates known as amyloid fibrils, which are less toxic (Figure 1). Much effort has therefore focused on identifying compounds that inhibit oligomer and fibril assembly or promote their disassembly (Hard and Lendel, 2012). However, progress has been hampered by a lack of detailed knowledge of the structure of amyloid oligomers and fibrils, particularly when they are bound to inhibitor molecules. Now, in *eLife*, Lin Jiang, David Eisenberg and co-workers at the University of California, Los Angeles, have designed several new compounds that reduce amyloid toxicity (Jiang et al., 2013). The starting point for their study was the high resolution structure of an Alzheimer’s peptide in an amyloid-like conformation bound to an inhibitor molecule (Landau et al., 2011).

**Figure 1.**
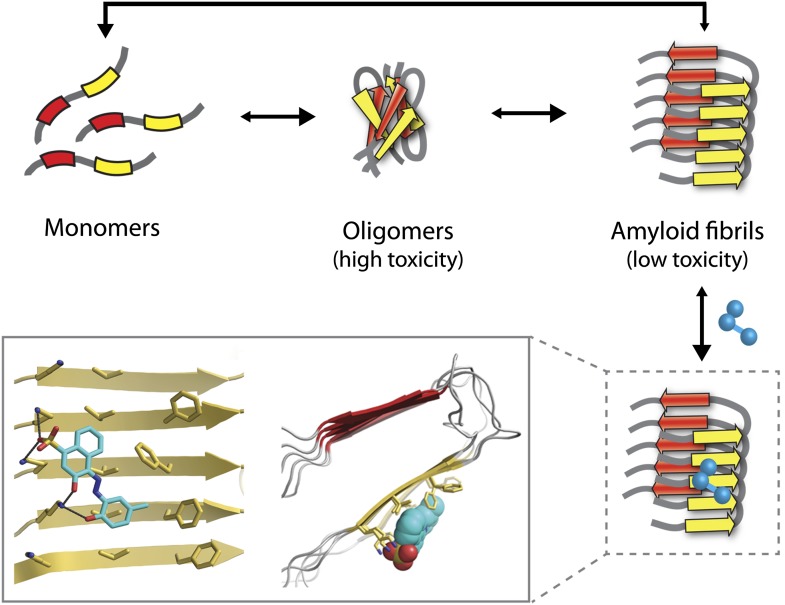
The Alzheimer’s peptide—initially soluble and benign when in monomeric form—assembles into oligomers, which are highly toxic to cells, and amyloid fibrils, which have low toxicity. Molecules that bind to and stabilize fibrils (example shown in blue) can inhibit amyloid toxicity by preventing the dissociation of fibrils into toxic oligomers, or into monomers that can reassemble into toxic oligomers. The yellow and red arrows in the fibrils represent individual β-strands. The inset shows an expanded view as seen from the front (left) and side (right). Inset images were adapted by Lin Jiang from Figure 1—figure supplement 2 of Jiang et al., 2013.

Structure-based drug design—in which high-resolution 3D structures of proteins are used to guide the design of compounds that bind tightly to disease-linked proteins and block their deleterious activity—is a key strategy for translating basic biomedical research into therapeutic compounds. More than 20 years ago, this approach was used to generate inhibitors of the HIV protease, and led to multiple antiviral drugs entering the clinic years earlier and at a much lower cost than would have otherwise been possible (Wlodawer and Erickson, 1993).

Unfortunately, the application of this design strategy to generating compounds that can inhibit the amyloid toxicity linked to Alzheimer’s and other neurodegenerative diseases has been frustratingly slow. One reason is that the methods used to solve high-resolution structures of most soluble proteins (X-ray crystallography and NMR) are not readily amenable to insoluble aggregates such as amyloid oligomers and fibrils. This has led to decades of painstaking research using alternative methods such as solid-state NMR to generate structural models of amyloid fibrils (Tycko, 2006), but the uncertainties in these structures limit their usefulness for drug design. Nevertheless, these tour de force studies have revealed several key structural features of amyloid fibrils. They have shown that monomers within fibrils are oriented perpendicular to the fibril axis and form stacks of β-strands in which the residues are aligned (Figure 1). These structural models also reveal large flat interfaces along the fibril axis, which appear more difficult to target with drugs than the pockets and grooves that are typically seen on the surface of soluble proteins such as enzymes.

Eisenberg and co-workers have now synergized a number of developments from their lab and others to perform the highest resolution structure-based drug design study to date for inhibitors of amyloid toxicity. In 2005, the Eisenberg lab crystallized a small amyloid-forming peptide from a yeast prion (an infectious misfolded protein) (Nelson et al., 2005). Its high-resolution atomic structure showed important similarities to lower resolution models of amyloid fibrils formed from other larger peptides and proteins. Since then, the UCLA researchers have crystallized a number of different amyloid-forming peptides (including multiple Alzheimer’s peptide fragments) (Sawaya et al., 2007). They have also solved the atomic structure of an Alzheimer’s peptide fragment in complex with a compound (‘orange-G’) that inhibits the toxic effects of amyloid on cells (Landau et al., 2011). Notably, orange-G binds to a flat interface along the fibril axis in a manner that appears to stabilize the fibrils (Figure 1, inset).

In this study, Eisenberg and co-workers leveraged these structural findings to identify other molecules that bind to the same flat interface on Alzheimer’s fibrils by screening large libraries of small molecules (>10,000) using novel computational methods. This approach yielded several compounds that bound to these fibrils with higher affinity than orange-G and also inhibited amyloid toxicity. Although the best inhibitors bound with modest (micromolar) affinity, it is likely that additional rounds of fibril-inhibitor co-crystallization and structural analysis will lead to the identification of inhibitors with even higher affinities.

One of the key discoveries in this study is that the new inhibitors do not prevent amyloid formation or trigger dissociation of fibrils. Instead, they appear to prevent toxicity by stabilizing Alzheimer’s fibrils and preventing them from dissociating into toxic oligomers or into monomers that could reassemble into toxic oligomers (Figure 1). This is consistent with the observation that fibrils associated with Parkinson’s disease can release toxic oligomers (Cremades et al., 2012) and that certain compounds can prevent Alzheimer’s amyloid toxicity without preventing fibril formation (Chen et al., 2010). However, the findings of the UCLA team provide some of the most direct evidence to date that small molecules can abolish the toxicity of mature fibrils solely by stabilizing them against dissociation. Nevertheless, this interesting finding will need to be studied in more detail to better understand how such inhibitors block amyloid toxicity.

This structure-based approach to drug design should catalyze future studies in multiple directions. First, the co-crystallization and X-ray structural analysis of additional small molecule inhibitors with Alzheimer’s peptide fragments will likely reveal additional binding sites, and may lead to the design of even more effective inhibitors. In addition, this approach should be readily applicable to amyloid fibrils linked to other disorders such as Parkinson’s and prion diseases because peptide fragments from their corresponding amyloid-forming proteins have already been crystallized in fibril-like conformations (Sawaya et al., 2007). Moreover, the insights from these studies will be valuable for guiding the design and selection of antibody and peptide inhibitors that have larger binding interfaces, and thus have the potential to bind with higher affinity and specificity than their small molecule counterparts. This important study has brightened the future for structure-based drug design by providing a path forward for generating inhibitors of one of the most complex and difficult-to-drug targets linked to human disease.
